# New species and records of Uropodina mites from Iran (Acari, Mesostigmata)

**DOI:** 10.3897/zookeys.600.8467

**Published:** 2016-06-22

**Authors:** Shahrooz Kazemi, Somayeh Abolghasemi

**Affiliations:** 1 Department of Biodiversity, Institute of Science and High Technology and Environmental Sciences, Graduate University of Advanced Technology, Kerman, Iran

**Keywords:** Trematuridae, soil-dwelling mites, Uropodidae, Uropodina

## Abstract

In this paper, a new species of the genus *Nenteria* Oudemans, 1915 is described on the basis of adult female and male specimens collected in soil and litter in parks in Kerman, southeastern Iran, and Tehran, northern Iran. A key to the Iranian species of *Nenteria* is also presented, and *Trachycilliba abantica* (Bal & Őzkan, 2007) is reported for the first time in Iran.

## Introduction

Mites of the infraorder Uropodina occur in forest soil and leaf litter, moss, rotting wood, dung, beach wrack, as well as in the nest of mammals, birds, and insect (Karg 1989, Wiśniewski and Hirschmann 1993, Błoszyk 1999, Mašán 2001, Lindquist et al. 2009). The classification of Uropodina, especially at higher level is not yet stable. For instance, the group was divided into five superfamilies (Protodinychoidea, Polyaspidoidea, Thinozerconoidea, Uropodoidea, Diarthrophalloidea) and 13 families by Lindquist et al. (2009), and into four superfamilies (Microgynioidea, Thinozerconoidea, Uropodoidea, Diarthrophalloidea) and 35 families by Beaulieu et al. (2011). Halliday (2016) catalogued the genera of Uropodina based on the classification of Beaulieu et al. (2011) and listed 300 genus-group names and their type species.

The Uropodoidea or “higher uropodines” (Evans 1972) is the largest superfamily of Uropodina and comprises over 2200 described species (Wiśniewski and Hirschmann 1993, Beaulieu et al. 2011). Although several attempts have been done to clarify the classification of uropodine mites, the boundaries of several groups of these mites, including the families and genera are not clear yet, such as the genus *Nenteria* that was erected by Oudemans (1915) with the type species of *Uropoda tropica* Oudemans, 1905. Some authors considered *Nenteria* as a genus of Uropodidae (e.g. Krantz and Aniscough 1990, Kadite and Petrova 1977), or placed it in the family Trematuridae (e.g. Karg 1989, Mašán 2001), or in the family Nenteriidae (e.g. Hirschmann 1979, Hirschmann and Wiśniewski 1985, Farrier and Hennessey 1993, Beaulieu et al. 2011, Kontschán 2012, 2014). Hirschmann and Wiśniewski (1985) stated that there were 109 described species of the genus *Nenteria* and divided them into eight species group, and later, in 1993, they mentioned 124 species in the genus (Wiśniewski and Hirschmann 1993).

Iranian mites of Uropodoidea (*sensu* Beaulieu et al., 2011) are poorly known. Until now, only 34 species of this superfamily have been reported in Iran, including three species of the genus *Nenteria* (*Nenteria breviunguiculata* Willmann, 1949; *Nenteria stammeri* Hirschmann & Z.-Nicol, 1962; *Nenteria stylifera* Berlese, 1904) and two species of *Trachycilliba* Berlese, 1903 (reported as members of *Neodiscopoma* Vitzthum, 1943: *Nenteria splendida* (Kramer, 1882) and *Nenteria persica* Kazemi & Kontschán, 2007) (Kazemi and Rajaei 2013, Arjomandi et al. 2013, Arjomandi and Kazemi 2014, in press, Kazemi et al. 2014, Kazemi and Kontschán 2014). The purpose of this paper is to describe a new species of *Nenteria* from Iran, present a key to the species of *Nenteria* occurring in the country, and report a species of *Trachycilliba* that is previously unknown in Iran.

## Material and methods

The mite specimens were extracted from soil of parks in Kerman and Tehran cities by Berlese-Tullgren funnels, cleared in Nesbitt’s fluid and then mounted in Hoyer’s medium on microscope slides.

Morphological observations, measurements and illustrations were made using a compound microscope equipped with differential interference contrast and phase contrast optical systems (Olympus BX51). Measurements are given in micrometers (μm). Dorsal shield length and width were respectively taken from the anterior to posterior shield margins along the midline and from the lateral margins at the broadest level. The length and width of the epigynal shield were measured from the anterior to posterior margins of the shield along the midline, and from the lateral margins of the shield at the broadest point, respectively. The length of the second cheliceral segment was measured from the base to the apex of the fixed digit, and its width at the broadest point. The length of the fixed cheliceral digit was taken from anterior level of nodus to the apex, and that of the movable digit from the base to apex. The legs length was taken from the base of the coxa to the apex of the tarsus, excluding the ambulacrum. Notation for setae on ventral and dorsal idiosoma mostly follows those of Lindquist and Evans (1965).

## Taxonomy

### Family Trematuridae

Taxon classificationAnimaliaMesostigmataTrematuridae

#### Note.

The genus *Nenteria* was placed in the family Nenteriidae by Hirschmann (1979), but he never published any diagnosis or description for the family. Although several subsequent authors referred to this family name (such as those mentioned in introduction), the family has never been described, and Nenteriidae remains a *nomen nudum* (Halliday, 2016). We therefore place *Nenteria* in the family Trematuridae, following Karg (1989) and Mašán (2001).

### Genus Nenteria

Taxon classificationAnimaliaMesostigmataNenteriidae

Oudemans, 1915

#### Type species.

*Uropoda tropica* Oudemans, 1905

#### Diagnosis.

The genus diagnosis of Mašán (2001) was followed.

### Nenteria bastanii sp. n.

Taxon classificationAnimaliaMesostigmataNenteriidae

http://zoobank.org/A4651411-6862-40CD-A76C-C8C9889986E9

Figures 1–5 , 6–10 , 11–16

#### Diagnosis

**(adult female and male).** Dorsal shield with 75 pairs of short and pectinate setae, and 2–4 unpaired median setae on propodosomal region. Marginal setae short and smooth, except pectinate setae *J5* and *Z5*. Female epigynal shield iron-shape, posteriorly reaching to mid-level of coxae IV, with an apical anterior spike, occasionally bifid at tip; shield surface smooth. Sternal setae *st1–5* smooth. Female ventral region behind epigynal shield bears 22–23 pairs of setae, including *Ad1*–2 and *st5*, and with 22 pairs in male, excluding *st4–5*, setae mostly short and acicular, except *Ad1–2*, *V7–8* longer and pectinate. Peritremes with a hook-like anterior extensions and without anterior projections to forward. Anterior edge of epistome bifid, without median hyaline flap. Cheliceral movable digit with a median tooth. Claws of leg I well-developed, sub-equal in size to other leg claws. Dorsal setae in femur, genu and tibia I of male mostly thicker than those in female.

#### Description.

***Female*** (n = 4). Idiosoma oval-shape, brown in color, 461–494 μm long, 336–346 μm wide.

**Figures 1–5.**
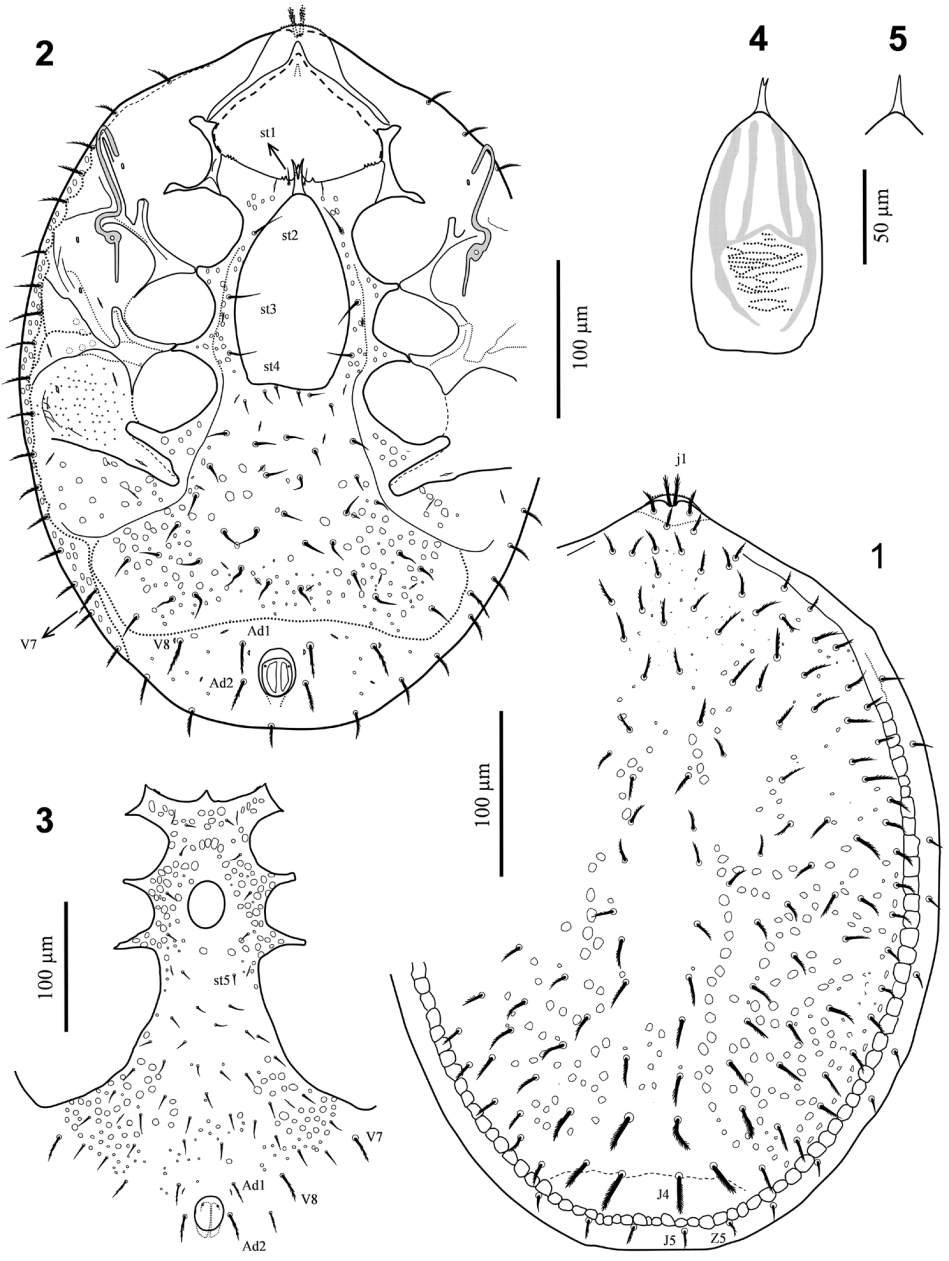
*Nenteria bastanii* sp. n. **1** Female, dorsal idiosoma **2** Female, ventral idiosoma **3** Male, holoventral region of idiosoma **4** Female, epigynal shield **5** Female, anterior spike of epigynal shield.

*Dorsal idiosoma* (Fig. 1). Dorsal shield surface ornamented with sparse sub-circular pits, more densely on opisthonotal region, median region of shield smooth. Dorsal shield setae 12–30 μm long, mostly slightly pectinate, *j1* (23–24 μm) apically plumose, posterior setae slightly thicker, longer and densely pectinate, *J4* longest (27–30 μm). Marginal shield narrow, bearing nine pairs of smooth and two pairs (*J5*, *Z5*) of pectinate setae, 12–16 μm long. With 20 pairs of submarginal plus one postanal seta, 18–23 μm long, situated subventrally.

*Venteral idiosoma* (Figs 2, 4–5, 9). Tritosternal base narrow, cylindrical, 28–31 μm long, 7–9 μm wide, with a pair of small denticles lateromedially and a pair apically; laciniae serrate, 26–31 μm long, apically trifid. Anterior margin of sternal region of holoventral shield with a median projection flanked by 1–2 small teeth; shield surface ornamented with sub-circular pits; sternal setae smooth, *st1* 10–11 μm long, adjacent, inserted near anterior margin of shield, *st2–4* 16–21 μm long, subequal. Epigynal shield iron-shape, 125–136 μm long (excluding anterior spike), 73–84 μm wide, with a relatively long anterior spike (18–20 μm), occasionally bifid at apex, shield surface smooth, posterior margin of shield truncate, ending at mid-level of coxae IV. Ventrianal region of holoventral shield with 22–23 pairs of setae, including *st5*, mostly smooth (12–18 μm), except pectinate para-anal setae *Ad1* (24–26 μm), *Ad2* (22–24 μm) and ventral setae *V7* (19–22 μm), *V8* (21–24 μm). Peritremes without anterior angle of 90°, poststigmatic section 22– 26 μm long. Anal opening oval-shape, 34–37 μm long, 24–28 μm wide. Surface between pedofossae III-IV with fine reticulate pattern.

**Figures 6–10.**
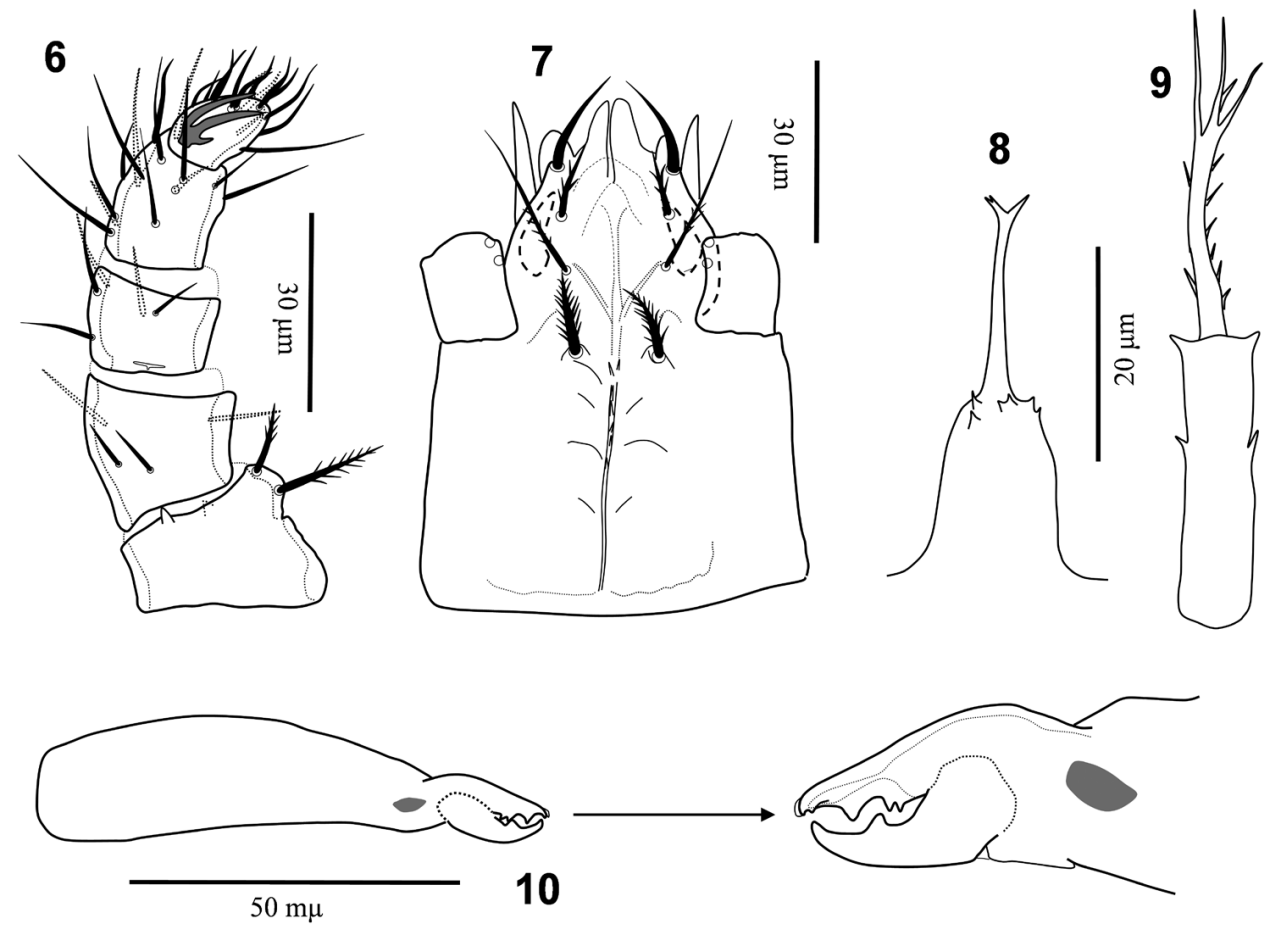
*Nenteria bastanii* sp. n. Female, **6** Palp **7** Subcapitulum **8** Epistome **9** Tritosternum **10** Chelicera.

*Gnathosoma* (Figs 6–7, 9–10). Base of epistome columnar and relatively wide, bearing some small denticulate outgrowths subapically, with a median narrow elongate projection, apically bifid (Fig. 8). Corniculi horn-like (14 μm), ventrally covered by subcapitulum; internal malae and paralaciniae smooth (Fig. 7). Hypostomal setae *h1* (16–18 μm) smooth, thickened, inserted on small protuberances, *h2* (11–15 μm) serrate, *h3* (26–28 μm) elongate, slender, with some barbs in basal half, capitular setae *h4* (14–16 μm) thickened, plumose. Deutosternal groove narrow, with few denticles behind capitular setae. Second segment of chelicera 81–83 μm long, 18–19 μm wide; fixed cheliceral digit 19–21 μm long, with a minute sub-apical denticle, and 2–3 teeth; movable digit 15–17 μm long, with a median tooth. Palp 74–80 μm long; palp chaetotaxy as “*Uropoda*-type” (*sensu* Evans, 1963): trochanter 2, femur 4, genu 5, tibia 14, tarsus 15; all setae smooth, except *v1*–*2* in trochanter pectinate.

**Figures 11–16.**
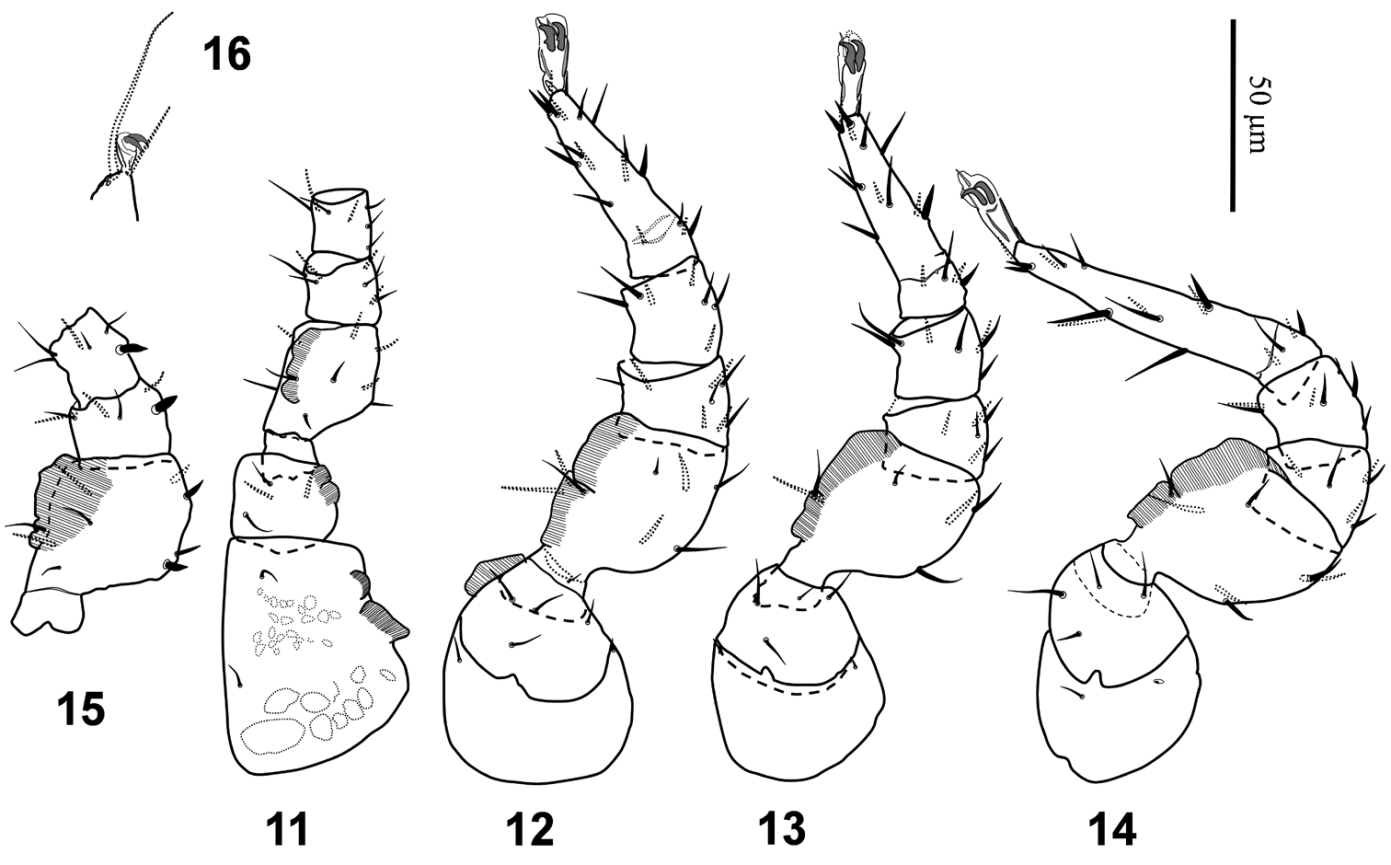
*Nenteria bastanii* sp. n. **11–14** Female, legs I-IV, posterolateral view **15** Male, leg I: femur-tibia **16** Female, apical region of tarsus I and pretarsus.

*Legs* (Figs 11–14, 16). Leg chaetotaxy “*Uropoda*-type” (*sensu* Evans, 1972). All legs with claws; leg I with a long sub-terminal seta (49–54 μm). Chitinus membrane present in coxae I, trochanter I-II, femura I-IV. Lengths of legs I-IV 242–256 μm, 185–198 μm, 192–214 μm and 245–247 μm, respectively. Lengths of femora I-IV 49–55 μm, 53–61 μm, 48–55 μm, 56–62 μm; genua I-IV 18–21 μm, 21–23 μm, 19–22 μm, 20–24 μm; tibiae I-IV 17–21 μm, 25–26 μm, 21–24 μm, 23–27 μm; tarsi I-IV 56–62 μm, 53–56 μm, 56–59 μm, 78–81 μm, respectively. Claws on tarsus I subequal to other legs claws (Fig. 16). Pretarsi I-IV 10–12 μm, 18–20 μm, 17–19 μm, 22–25 μm, respectively. Leg setae mostly narrow, needle-like and short, as figures11–14.

***Male*** (n = 3). Idiosoma 440–464 μm long, 343–357 μm wide.

*Dorsal shield*. Dorsal shield characters similar to those in female. Dorsal shield setae 9–28 long, *j1* 22–24 μm, *J4* 24–28 μm long. Marginal setae 9–14 μm long; submarginal and postanal setae 17–22 μm long.

*Ventral shield*. Sternal setae smooth, *st1* 10–12 μm, *st2* 17–19 μm, *st3* 13–17 μm, *st4*–*5* 12–15 μm long. Operculum oval-shape, 38–42 μm long, 25–33 μm wide. Ventrianal region with 20 pairs of simple, smooth setae (12–15 μm), except pectinate setae *Ad1*–*2* (22–25 μm), *V7–8* (20–23 μm).

*Gnathosoma*. Hypostomal seta shape similar to those in female, *h1* 14–16 μm, *h2* 12–15 μm, *h3* 29–31 μm, *h4* 14 μm long. Corniculi horn- like (14 μm). Fixed and movable digit of chelicerae similar to female, fixed digit 19–20 μm long, movable digit 15–16 μm long. Palp 73 μm long.

*Legs* (Fig. 15). Leg chaetotaxy and chitinous membrane similar to those in female, but dorsal setae in femur I, genu I and tibia I mostly thickened in male. Lengths of legs I-IV 232–273 μm, 213–248 μm, 200–212 μm, 236–251 μm, respectively. Lengths of femora I-IV 44–51 μm, 57–61 μm, 56–60 μm,56–62 μm; genua I-IV 19–20 μm, 20–25 μm, 21–25 μm, 23–28 μm; tibiae I-IV 18–20 μm, 20–25 μm, 21–25 μm, 24–27 μm; tarsi I-IV 55–59 μm, 52–56 μm, 55–59 μm, 82–87 μm, respectively. Pretarsus I 9–12 μm long, pretarsi II-IV 18–22 μm long. Apical long seta on tarsus I 51–56 μm long.

#### Material examined.

Holotype: female, southeastern Iran, Kerman Province, Kerman, Shora Park (30°29'84"N; 57°07'10"E), 1761 m a.s.l., from soil, 28 Sept 2015, coll. S. Kazemi, deposited in Acarological Collection, Institute of Science and High Technology and Environmental Sciences, Graduate University of Advanced Technology, Kerman, Iran (ACISTE). Paratypes: four females and two males with same collection data, deposited in ACISTE; three females and one male, Kerman, Pardis Park (30°29'81"N; 57°07'01"E), 1760 m a.s.l., from soil, 16 Aug 2015, coll. S. Abolghasemi, deposited in ACISTE; two females and one male collected in Tehran Province, Tehran, Taleghani Park (35°45'13"N; 51°25'26"E), 1424 m a.s.l., from soil and litter, 15 Sept 2015, coll. S. Kazemi, deposited in ACISTE.

#### Etymology.

The species is named in honor of the famous current Iranian historian, poet, translator and writer who died in March 2014, Prof. Mohammad Bastani Parizi.

#### Remarks.

The new species can be easily distinguished from other described species of the genus by presence of 22–23 pairs of setae in ventral region of the holoventral shield behind the epigynal plate (including setae *st5*) in female and 22 pairs in male (excluding *st4–5*).

### Key to the known Iranian species of *Nenteria* (female)

**Table:** 

1	Peritreme with an anterior projection, curved to the front in a 90° angle	***Nenteria stylifera* Berlese, 1904**
–	Peritremes without anterior projections to forward	**2**
2	Opisthogastric region behind epigynal shield with 22–23 pairs of setae; epigynal shield with an apical narrow spike	***Nenteria bastanii* sp. n.**
–	Opisthogastric region behind epigynal shield with 9–10 pairs of setae; epigynal shield without narrow apical spike	**3**
3	Surface between pedofossae with oval pits	***Nenteria stammeri* Hirschmann & Z- Nicol, 1961**
–	Surface between pedofossae with reticulate pattern	***Nenteria breviunguiculata* Willmann, 1949**

### Genus Trachycilliba

Taxon classificationAnimaliaMesostigmataTrematuridae

Berlese, 1903

#### Type species.

*Uropoda splendida* Kramer, 1882.

### Trachycilliba abantica

Taxon classificationAnimaliaMesostigmataTrematuridae

(Bal & Özkan, 2007)

Uropoda abantica Bal & Özkan, 2007: 43.
Neodiscopoma abantica .—Kontschán, 2013: 118.
Trachycilliba abantica .—**new combination**.

#### Studied materials.

One female and one male specimens from soil and litter in the Ecological Garden of Nowshahr (51°57'50"N; 40°55'74"E), Mazandaran Province, northern Iran, altitude 30 m a.s.l., 10 June 2014, deposited in ACISTE.

#### Note.

Bal and Özkan (2007) described this species as a member of *Uropoda* Latreille, 1808 *sensu lato* from Turkey. Kontschán (2013) reported it from Greece, and he transferred it to the genus *Neodiscopoma* Vitzthum, 1942, based on the species morphological characters, such as a dorsal shield with a strongly sclerotized median region elevated from other parts; and posteromarginal setae inserted on separate, individual platelets. The genera *Neodiscopoma* and *Trachycilliba* have a same type species, *Uropoda splendida* Kramer, 1882, therefore we placed the species within *Trachycilliba*. Herein, we report *Trachycilliba abantica* from the Ecological Garden of Nowshahr in northern Iran near the Caspian Sea. This represents the third report of this species in the world.

## Supplementary Material

XML Treatment for Trematuridae

XML Treatment for Nenteria

XML Treatment for Nenteria bastanii

XML Treatment for Trachycilliba

XML Treatment for Trachycilliba abantica
